# Editorial: Scaling-up health-IT—sustainable digital health implementation and diffusion

**DOI:** 10.3389/fdgth.2024.1296495

**Published:** 2024-04-15

**Authors:** Hannes Schlieter, Kai Gand, Lisa A. Marsch, Wai Sze Chan, Tobias Kowatsch

**Affiliations:** ^1^ Research Group Digital Health, Technische Universität Dresden, Dresden, Germany; ^2^ Geisel School of Medicine at Dartmouth, Dartmouth College, Hanover, NH, United States; ^3^ Department of Psychology, University of Hong Kong, Hong Kong, Hong Kong SAR, China; ^4^ Institute for Implementation Science in Health Care, University of Zurich, Zurich, Switzerland; ^5^ School of Medicine, University of St. Gallen, St. Gallen, Switzerland; ^6^ Centre for Digital Health Interventions, Department of Management, Technology, and Economics, ETH Zurich, Zurich, Switzerland

**Keywords:** digital health, eHealth, implementation strategy, implementation research, transformation of traditional care processes, dissemination and adoption strategies

**Editorial on the Research Topic** Scaling-up health-IT—sustainable digital health implementation and diffusion

Digital health technologies [DHTs; (1)] are increasingly employed by providers and patients for prevention, diagnosis, management, and treatment. For example, digital diagnostics improve the efficiency of clinical workflows (2), or patients receive prescriptions for digital therapeutics (3). However, the question of how DHTs can be scaled up efficiently and sustainably remains challenging and has not yet been sufficiently addressed (4, 5). The “time-to-market” for evidence-based practice can still be up to 15 years (6), and digital health startups have the highest failure rate of 98% across industries (7).

**Figure 1:**
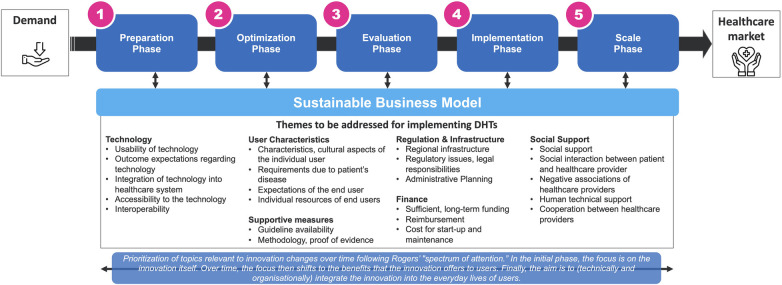
How to build sustainable digital health innovations?, inspired by (4, 8, 9), referring to the “spectrum of attention” for the diffusion of innovations by (10). This figure has been designed using images from Flaticon.com.

Figure 1 highlights relevant barriers that precisely address the challenges for sustainably building a DHT outlined above. Details can be found in the underlying references. Procedural aspects of the implementation of DHTs can be found in more detail, for example, in Van Gemert-Pijnen et al. (11). We deem regular evaluation, particularly phases 1–3 in Figure 1, as crucial for potentially enabling evaluation-based adjustments to DHTs to overcome barriers. However, the detailed discussion of these procedural aspects is beyond this scope. Additionally, quantifying implementation outcomes remains unclear due to poorly defined and operationalized constructs like adoption and sustainability, with a notable absence of standardized measures (12, 13). So, in principle, different implementation outcomes are relevant at different phases of implementation. While regulatory affairs and user characteristics are usually prime during preparation, technical interoperability could be part of optimization. As such innovation processes are embedded in different innovation ecosystems, Figure 1 illustrates the main categories and pathways to proper diffusion. Also, Figure 1 emphasizes the importance of identifying target populations and their needs (demand preparation). This includes the barriers to implementing DHTs, such as technology usability, user characteristics, regulation (Medical Device or General Data Protection Regulation) and (IT or clinical) infrastructure, social support (family background, friends' networks), and cultural aspects (e.g., different understandings of care). To build sustainable DHTs, it's essential to enhance technology, ensure usability, deploy, and assess impact in target settings. Evaluation informs iterative improvements, crucial for scaling. Challenges like funding, infrastructure, regulatory support, user engagement, and sustainability need addressing to boost DHT scaling success. However, the prioritization of topics relevant to innovation changes within a “spectrum of attention”. Firstly, the focus is on the innovation itself. Over time, the focus then shifts to the benefits that the innovation offers users. Finally, the aim is to integrate the innovation into the everyday lives of users (10).

The research topic explores some central issues and opportunities in scaling up DHTs, with a focus on early-stage DHTs with high potential and focusing on some of the themes mentioned.

Rusch et al. and Azevedo et al. highlight the *importance of usability and acceptability for successfully scaling up DHTs*. This is about designing and implementing user-centered DHTs by engaging patients, providers, policymakers, and payers, ensuring the technologies meet real-world needs and are sustainably and equitably implemented. Also, Azevedo et al. outline that different implementation outcomes are relevant at different phases of implementation (as touched above). From a technical usability perspective, Sieber et al. note that while personalized, face-to-face support in DHTs boosts retention, it raises scalability challenges due to staffing demands. They recommend a socio-technical approach that balances personalization with scalability from the outset. Aronson et al. and Otto et al. highlight the need for *scaling-up strategies to be tailored*, accounting for varied populations and settings. This is due to the differing effectiveness and acceptability of DHTs based on the target population's needs and the implementation context. Adaptations may include changes to the DHT, its delivery, available support resources, or to meet the cultural and linguistic needs of the target population. Cultural adaptation ensures that the DHT is respectful of the target's values and beliefs. Particularly, Hazra-Ganju et al. highlight the importance of *localization and cultural adaptation* when scaling up DHTs in resource-limited settings. This is because DHTs that are not tailored to the local context are less likely to be effective and sustainable. Looking at a program/meso level, Williams et al. introduce a holistic care concept called Parsley Health to deliver preventive measures for chronic conditions. A feasible and acceptable scaling up was shown, with symptom severity declining and reasonable satisfaction with the services. To this end, Castro et al. are planning a study to explore the sustainable development of a low-cost, smartphone-based intervention with a conversational agent for lifestyle support, potentially revealing general health benefits and scalability. Otto et al., stress the importance of *community engagement in the success and scaling of DHTs*, highlighting the community's role in advocating for DHT usage and adoption. This includes community involvement in design, development, implementation, and promotion of DHTs.

The research topic emphasizes the necessity for a systematic, evidence-based approach to scale up DHTs, highlighting the current fragmented and ad-hoc methods that result in ineffective, unsustainable, or inequitable DHT implementations. It sheds light on critical aspects such as user-centered design, collaborative development, tailored strategies for scaling up, localization with cultural adaptation, and community engagement as key to successful DHT adoption. Addressing these areas will enhance DHT adoption and healthcare improvement. To this end, future research on DHTs should focus on improving clinical outcomes in the long term in the most cost-efficient way. Additionally, research is needed to address regulatory challenges, enhancing trust, transparency, adoption rates, and interoperability. Sustainable reimbursement models are required for the continuous maintenance and iterative improvement of DHTs, mainly when artificial intelligence is used and fed by healthcare data that is increasingly available. It is needed study the potential and boundaries of generative language models since they promise high expectations on the time to market of DHTs but also put risks regarding patient safety, evidence, and reliability to the market. We also call for contributions investigating barriers and drivers for typical stakeholders such as payers, physicians, nurses, and patients.
